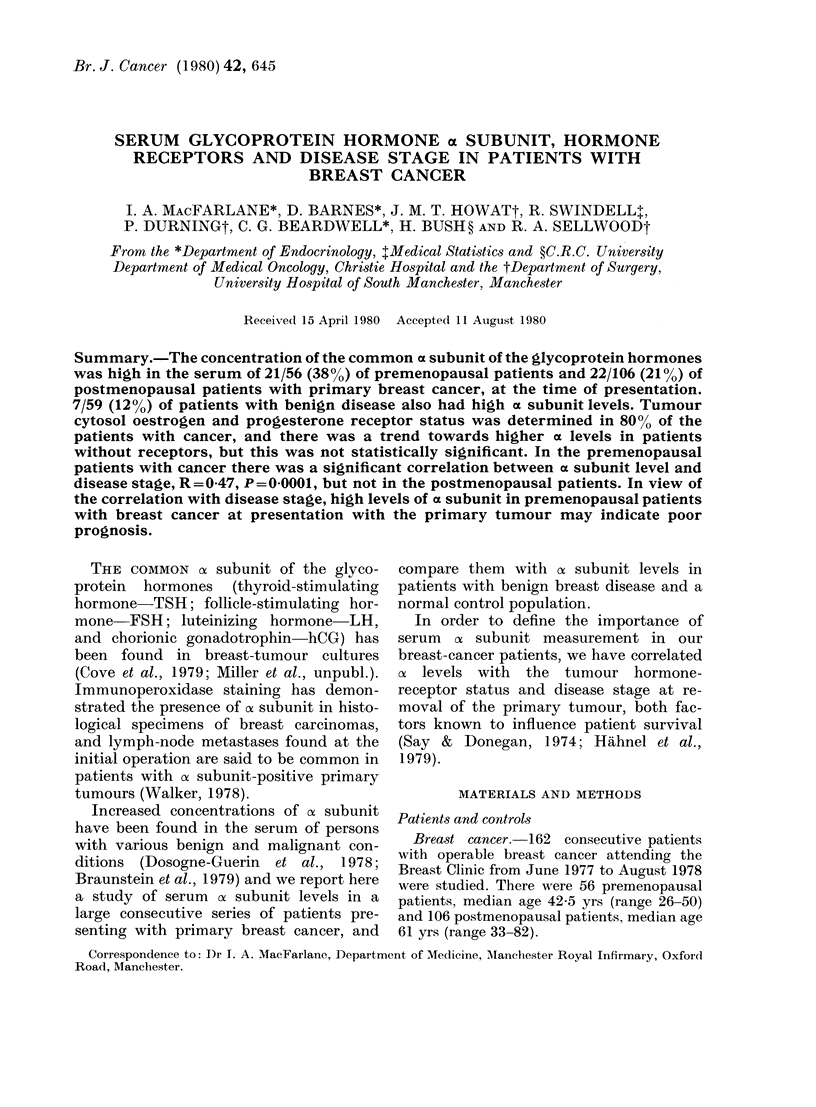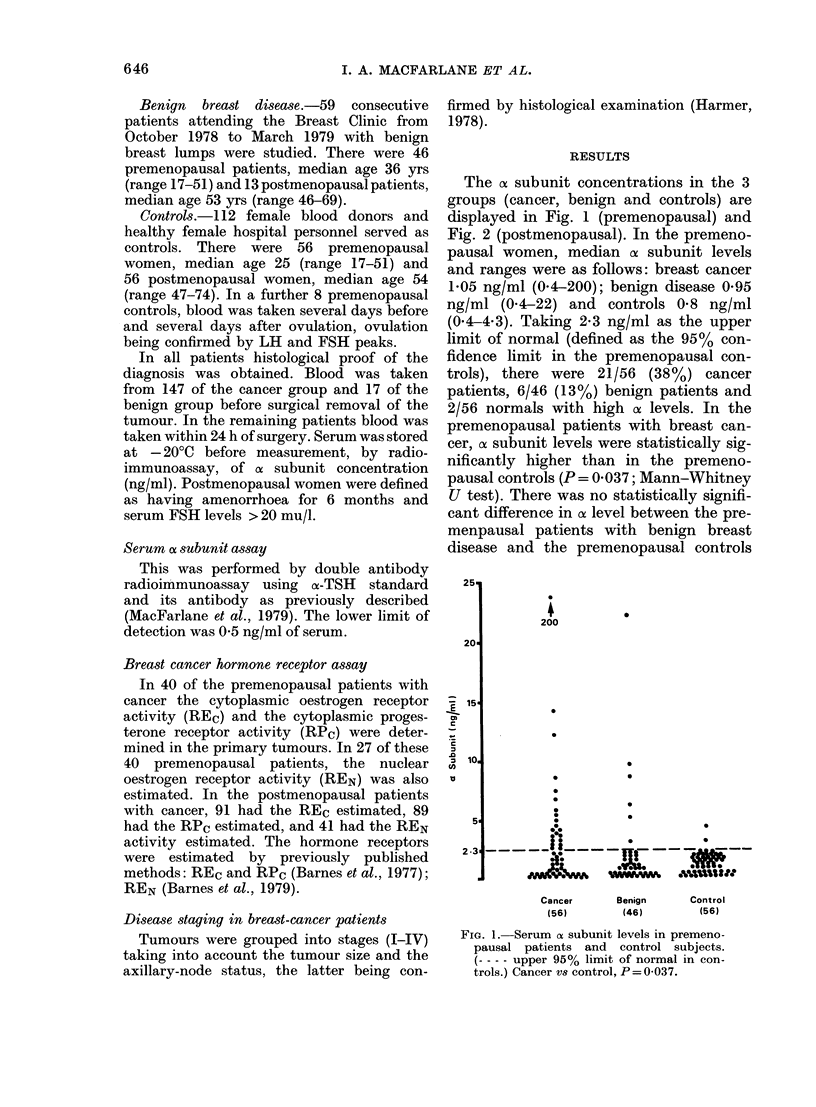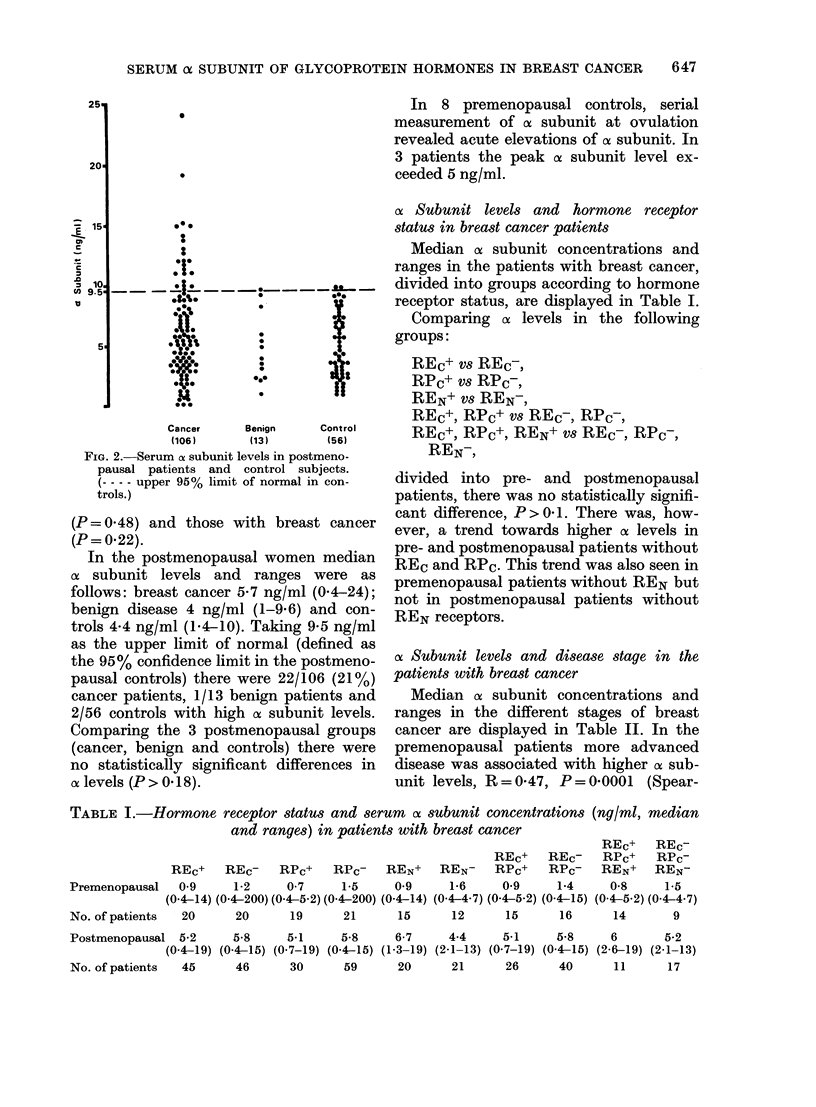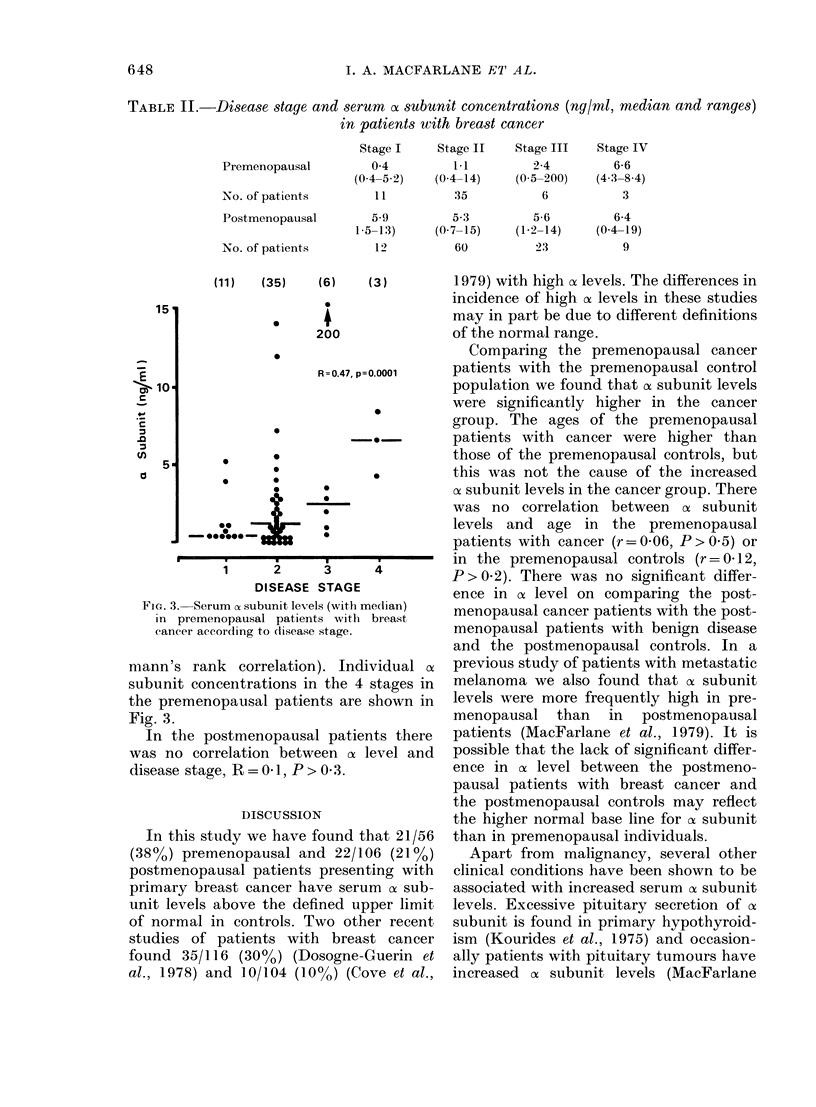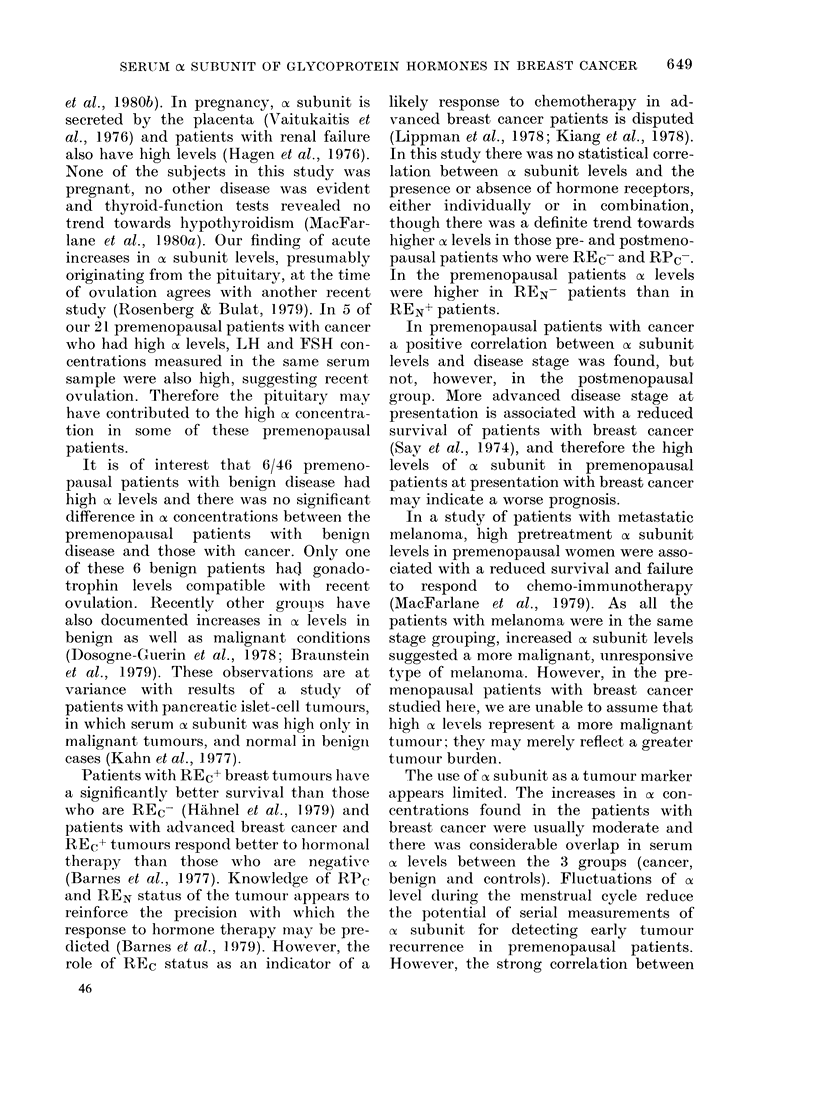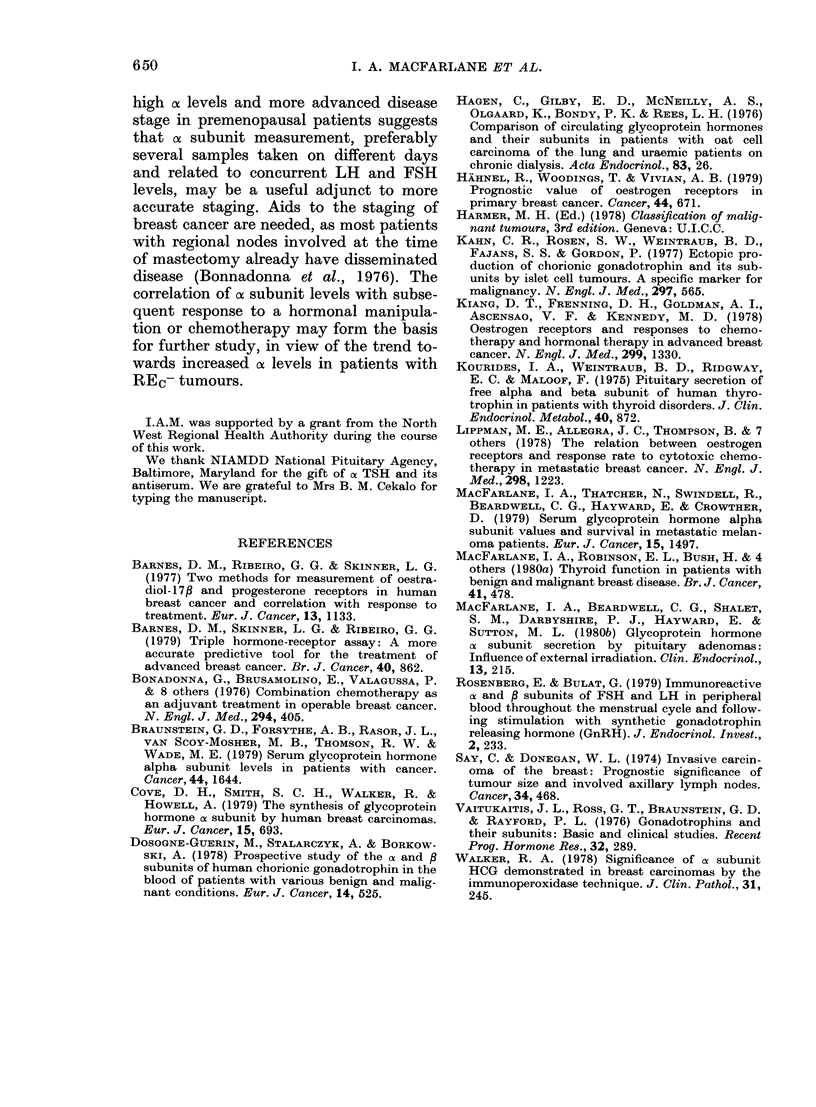# Serum glycoprotein hormone alpha subunit, hormone receptors and disease stage in patients with breast cancer.

**DOI:** 10.1038/bjc.1980.297

**Published:** 1980-11

**Authors:** I. A. MacFarlane, D. Barnes, J. M. Howat, R. Swindell, P. Durning, C. G. Beardwell, H. Bush, R. A. Sellwood

## Abstract

The concentration of the common alpha subunit of the glycoprotein hormones was high in the serum of 21/56 (38%) of premenopausal patients and 22/106 (21%) of postmenopausal patients with primary breast cancer, at the time of presentation. 7/59 (12%) of patients with benign disease also had high alpha subunit levels. Tumour cytosol oestrogen and progesterone receptor status was determined in 80% of the patients with cancer, and there was a trend towards higher alpha levels in patients without receptors, but this was not statistically significant. In the premenopausal patients with cancer there was a significant correlation between alpha subunit level and disease stage, R = 0.47, P = 0.0001, but not in the postmenopausal patients. In view of the correlation with disease stage, high levels of alpha subunit in premenopausal patients with breast cancer at presentation with the primary tumour may indicate poor prognosis.


*Br. J. Cancer* (1980) **42**, 645

### SERUM GLYCOPROTEIN HORMONE α SUBUNIT, HORMONE RECEPTORS AND DISEASE STAGE IN PATIENTS WITH BREAST CANCER

I. A. MacFARLANE*, D. BARNES*, J. M. T. HOWAT†, R. SWINDELL‡, P. DURNING†, C. G. BEARDWELL*, H. BUSH§ AND R. A. SELLWOOD†

*From the *Department of Endocrinology, ‡Medical Statistics and §C.R.C. University Department of Medical Oncology, Christie Hospital and the †Department of Surgery, University Hospital of South Manchester, Manchester*

Received 15 April 1980 Accepted 11 August 1980

**Summary.—The concentration of the common α subunit of the glycoprotein hormones was high in the serum of 21/56 (38%) of premenopausal patients and 22/106 (21%) of postmenopausal patients with primary breast cancer, at the time of presentation. 7/59 (12%) of patients with benign disease also had high α subunit levels. Tumour cytosol oestrogen and progesterone receptor status was determined in 80% of the patients with cancer, and there was a trend towards higher α levels in patients without receptors, but this was not statistically significant. In the premenopausal patients with cancer there was a significant correlation between α subunit level and disease stage, R=0·47, *P*=0·0001, but not in the postmenopausal patients. In view of the correlation with disease stage, high levels of α subunit in premenopausal patients with breast cancer at presentation with the primary tumour may indicate poor prognosis.**

THE COMMON α subunit of the glycoprotein hormones (thyroid-stimulating hormone—TSH; follicle-stimulating hormone—FSH; luteinizing hormone—LH, and chorionic gonadotrophin—hCG) has been found in breast-tumour cultures (Cove *et al.*, 1979; Miller *et al.*, unpubl.). Immunoperoxidase staining has demonstrated the presence of α subunit in histological specimens of breast carcinomas, and lymph-node metastases found at the initial operation are said to be common in patients with α subunit-positive primary tumours (Walker, 1978).

Increased concentrations of α subunit have been found in the serum of persons with various benign and malignant conditions (Dosogne-Guerin *et al.*, 1978; Braunstein *et al.*, 1979) and we report here a study of serum α subunit levels in a large consecutive series of patients presenting with primary breast cancer, and compare them with α subunit levels in patients with benign breast disease and a normal control population.

In order to define the importance of serum α subunit measurement in our breast-cancer patients, we have correlated α levels with the tumour hormone-receptor status and disease stage at removal of the primary tumour, both factors known to influence patient survival (Say & Donegan, 1974; Hähnel *et al.*, 1979).

### MATERIALS AND METHODS

### *Patients and controls*

*Breast cancer.*—162 consecutive patients with operable breast cancer attending the Breast Clinic from June 1977 to August 1978 were studied. There were 56 premenopausal patients, median age 42·5 yrs (range 26–50) and 106 postmenopausal patients, median age 61 yrs (range 33–82).

Correspondence to: Dr I. A. MacFarlane, Department of Medicine, Manchester Royal Infirmary, Oxford Road, Manchester.

*Benign breast disease.*—59 consecutive patients attending the Breast Clinic from October 1978 to March 1979 with benign breast lumps were studied. There were 46 premenopausal patients, median age 36 yrs (range 17–51) and 13 postmenopausal patients, median age 53 yrs (range 46–69).

*Controls.*—112 female blood donors and healthy female hospital personnel served as controls. There were 56 premenopausal women, median age 25 (range 17–51) and 56 postmenopausal women, median age 54 (range 47–74). In a further 8 premenopausal controls, blood was taken several days before and several days after ovulation, ovulation being confirmed by LH and FSH peaks.

In all patients histological proof of the diagnosis was obtained. Blood was taken from 147 of the cancer group and 17 of the benign group before surgical removal of the tumour. In the remaining patients blood was taken within 24 h of surgery. Serum was stored at −20°C before measurement, by radioimmunoassay, of $\alpha$ subunit concentration (ng/ml). Postmenopausal women were defined as having amenorrhoea for 6 months and serum FSH levels >20 mu/l.

### *Serum $\alpha$ subunit assay*

This was performed by double antibody radioimmunoassay using $\alpha$-TSH standard and its antibody as previously described (MacFarlane *et al.*, 1979). The lower limit of detection was 0·5 ng/ml of serum.

### *Breast cancer hormone receptor assay*

In 40 of the premenopausal patients with cancer the cytoplasmic oestrogen receptor activity ($RE_C$) and the cytoplasmic progesterone receptor activity ($RP_C$) were determined in the primary tumours. In 27 of these 40 premenopausal patients, the nuclear oestrogen receptor activity ($RE_N$) was also estimated. In the postmenopausal patients with cancer, 91 had the $RE_C$ estimated, 89 had the $RP_C$ estimated, and 41 had the $RE_N$ activity estimated. The hormone receptors were estimated by previously published methods: $RE_C$ and $RP_C$ (Barnes *et al.*, 1977); $RE_N$ (Barnes *et al.*, 1979).

### *Disease staging in breast-cancer patients*

Tumours were grouped into stages (I–IV) taking into account the tumour size and the axillary-node status, the latter being confirmed by histological examination (Harmer, 1978).

### RESULTS

The $\alpha$ subunit concentrations in the 3 groups (cancer, benign and controls) are displayed in Fig. 1 (premenopausal) and Fig. 2 (postmenopausal). In the premenopausal women, median $\alpha$ subunit levels and ranges were as follows: breast cancer 1·05 ng/ml (0·4–200); benign disease 0·95 ng/ml (0·4–22) and controls 0·8 ng/ml (0·4–4·3). Taking 2·3 ng/ml as the upper limit of normal (defined as the 95% confidence limit in the premenopausal controls), there were 21/56 (38%) cancer patients, 6/46 (13%) benign patients and 2/56 normals with high $\alpha$ levels. In the premenopausal patients with breast cancer, $\alpha$ subunit levels were statistically significantly higher than in the premenopausal controls ($P = 0·037$; Mann–Whitney $U$ test). There was no statistically significant difference in $\alpha$ level between the premenpausal patients with benign breast disease and the premenopausal controls

FIG. 1.—Serum $\alpha$ subunit levels in premenopausal patients and control subjects. (- - - - upper 95% limit of normal in controls.) Cancer *vs* control, $P = 0·037$.

FIG. 2.—Serum α subunit levels in postmenopausal patients and control subjects. (- - - - upper 95% limit of normal in controls.)

($P = 0{\cdot}48$) and those with breast cancer ($P = 0{\cdot}22$).

In the postmenopausal women median α subunit levels and ranges were as follows: breast cancer 5·7 ng/ml (0·4–24); benign disease 4 ng/ml (1–9·6) and controls 4·4 ng/ml (1·4–10). Taking 9·5 ng/ml as the upper limit of normal (defined as the 95% confidence limit in the postmenopausal controls) there were 22/106 (21%) cancer patients, 1/13 benign patients and 2/56 controls with high α subunit levels. Comparing the 3 postmenopausal groups (cancer, benign and controls) there were no statistically significant differences in α levels ($P > 0{\cdot}18$).

In 8 premenopausal controls, serial measurement of α subunit at ovulation revealed acute elevations of α subunit. In 3 patients the peak α subunit level exceeded 5 ng/ml.

*α Subunit levels and hormone receptor status in breast cancer patients*

Median α subunit concentrations and ranges in the patients with breast cancer, divided into groups according to hormone receptor status, are displayed in Table I.

Comparing α levels in the following groups:

$RE_C^+$ *vs* $RE_C^-$,
$RP_C^+$ *vs* $RP_C^-$,
$RE_N^+$ *vs* $RE_N^-$,
$RE_C^+$, $RP_C^+$ *vs* $RE_C^-$, $RP_C^-$,
$RE_C^+$, $RP_C^+$, $RE_N^+$ *vs* $RE_C^-$, $RP_C^-$, $RE_N^-$, divided into pre- and postmenopausal patients, there was no statistically significant difference, $P > 0{\cdot}1$. There was, however, a trend towards higher α levels in pre- and postmenopausal patients without $RE_C$ and $RP_C$. This trend was also seen in premenopausal patients without $RE_N$ but not in postmenopausal patients without $RE_N$ receptors.

*α Subunit levels and disease stage in the patients with breast cancer*

Median α subunit concentrations and ranges in the different stages of breast cancer are displayed in Table II. In the premenopausal patients more advanced disease was associated with higher α subunit levels, $R = 0{\cdot}47$, $P = 0{\cdot}0001$ (Spear-

TABLE I.—*Hormone receptor status and serum α subunit concentrations (ng/ml, median and ranges) in patients with breast cancer*

| | $RE_C^+$ | $RE_C^-$ | $RP_C^+$ | $RP_C^-$ | $RE_N^+$ | $RE_N^-$ | $RE_C^+$ $RP_C^+$ | $RE_C^-$ $RP_C^-$ | $RE_C^+$ $RP_C^+$ $RE_N^+$ | $RE_C^-$ $RP_C^-$ $RE_N^-$ |
|---|---|---|---|---|---|---|---|---|---|---|
| Premenopausal | 0·9 (0·4–14) | 1·2 (0·4–200) | 0·7 (0·4–5·2) | 1·5 (0·4–200) | 0·9 (0·4–14) | 1·6 (0·4–4·7) | 0·9 (0·4–5·2) | 1·4 (0·4–15) | 0·8 (0·4–5·2) | 1·5 (0·4–4·7) |
| No. of patients | 20 | 20 | 19 | 21 | 15 | 12 | 15 | 16 | 14 | 9 |
| Postmenopausal | 5·2 (0·4–19) | 5·8 (0·4–15) | 5·1 (0·7–19) | 5·8 (0·4–15) | 6·7 (1·3–19) | 4·4 (2·1–13) | 5·1 (0·7–19) | 5·8 (0·4–15) | 6 (2·6–19) | 5·2 (2·1–13) |
| No. of patients | 45 | 46 | 30 | 59 | 20 | 21 | 26 | 40 | 11 | 17 |

TABLE II.—*Disease stage and serum α subunit concentrations (ng/ml, median and ranges) in patients with breast cancer*

| | Stage I | Stage II | Stage III | Stage IV |
|---|---|---|---|---|
| Premenopausal | 0·4 (0·4–5·2) | 1·1 (0·4–14) | 2·4 (0·5–200) | 6·6 (4·3–8·4) |
| No. of patients | 11 | 35 | 6 | 3 |
| Postmenopausal | 5·9 1·5–13) | 5·3 (0·7–15) | 5·6 (1·2–14) | 6·4 (0·4–19) |
| No. of patients | 12 | 60 | 23 | 9 |

FIG. 3.—Serum α subunit levels (with median) in premenopausal patients with breast cancer according to disease stage.

mann's rank correlation). Individual α subunit concentrations in the 4 stages in the premenopausal patients are shown in Fig. 3.

In the postmenopausal patients there was no correlation between α level and disease stage, $R = 0{\cdot}1$, $P > 0{\cdot}3$.

### DISCUSSION

In this study we have found that 21/56 (38%) premenopausal and 22/106 (21%) postmenopausal patients presenting with primary breast cancer have serum α subunit levels above the defined upper limit of normal in controls. Two other recent studies of patients with breast cancer found 35/116 (30%) (Dosogne-Guerin *et al.*, 1978) and 10/104 (10%) (Cove *et al.*, 1979) with high α levels. The differences in incidence of high α levels in these studies may in part be due to different definitions of the normal range.

Comparing the premenopausal cancer patients with the premenopausal control population we found that α subunit levels were significantly higher in the cancer group. The ages of the premenopausal patients with cancer were higher than those of the premenopausal controls, but this was not the cause of the increased α subunit levels in the cancer group. There was no correlation between α subunit levels and age in the premenopausal patients with cancer ($r = 0{\cdot}06$, $P > 0{\cdot}5$) or in the premenopausal controls ($r = 0{\cdot}12$, $P > 0{\cdot}2$). There was no significant difference in α level on comparing the postmenopausal cancer patients with the postmenopausal patients with benign disease and the postmenopausal controls. In a previous study of patients with metastatic melanoma we also found that α subunit levels were more frequently high in premenopausal than in postmenopausal patients (MacFarlane *et al.*, 1979). It is possible that the lack of significant difference in α level between the postmenopausal patients with breast cancer and the postmenopausal controls may reflect the higher normal base line for α subunit than in premenopausal individuals.

Apart from malignancy, several other clinical conditions have been shown to be associated with increased serum α subunit levels. Excessive pituitary secretion of α subunit is found in primary hypothyroidism (Kourides *et al.*, 1975) and occasionally patients with pituitary tumours have increased α subunit levels (MacFarlane

*et al.*, 1980*b*). In pregnancy, α subunit is secreted by the placenta (Vaitukaitis *et al.*, 1976) and patients with renal failure also have high levels (Hagen *et al.*, 1976). None of the subjects in this study was pregnant, no other disease was evident and thyroid-function tests revealed no trend towards hypothyroidism (MacFarlane *et al.*, 1980*a*). Our finding of acute increases in α subunit levels, presumably originating from the pituitary, at the time of ovulation agrees with another recent study (Rosenberg & Bulat, 1979). In 5 of our 21 premenopausal patients with cancer who had high α levels, LH and FSH concentrations measured in the same serum sample were also high, suggesting recent ovulation. Therefore the pituitary may have contributed to the high α concentration in some of these premenopausal patients.

It is of interest that 6/46 premenopausal patients with benign disease had high α levels and there was no significant difference in α concentrations between the premenopausal patients with benign disease and those with cancer. Only one of these 6 benign patients had gonadotrophin levels compatible with recent ovulation. Recently other groups have also documented increases in α levels in benign as well as malignant conditions (Dosogne-Guerin *et al.*, 1978; Braunstein *et al.*, 1979). These observations are at variance with results of a study of patients with pancreatic islet-cell tumours, in which serum α subunit was high only in malignant tumours, and normal in benign cases (Kahn *et al.*, 1977).

Patients with $RE_C^+$ breast tumours have a significantly better survival than those who are $RE_C^-$ (Hähnel *et al.*, 1979) and patients with advanced breast cancer and $RE_C^+$ tumours respond better to hormonal therapy than those who are negative (Barnes *et al.*, 1977). Knowledge of $RP_C$ and $RE_N$ status of the tumour appears to reinforce the precision with which the response to hormone therapy may be predicted (Barnes *et al.*, 1979). However, the role of $RE_C$ status as an indicator of a likely response to chemotherapy in advanced breast cancer patients is disputed (Lippman *et al.*, 1978; Kiang *et al.*, 1978). In this study there was no statistical correlation between α subunit levels and the presence or absence of hormone receptors, either individually or in combination, though there was a definite trend towards higher α levels in those pre- and postmenopausal patients who were $RE_C^-$ and $RP_C^-$. In the premenopausal patients α levels were higher in $RE_N^-$ patients than in $RE_N^+$ patients.

In premenopausal patients with cancer a positive correlation between α subunit levels and disease stage was found, but not, however, in the postmenopausal group. More advanced disease stage at presentation is associated with a reduced survival of patients with breast cancer (Say *et al.*, 1974), and therefore the high levels of α subunit in premenopausal patients at presentation with breast cancer may indicate a worse prognosis.

In a study of patients with metastatic melanoma, high pretreatment α subunit levels in premenopausal women were associated with a reduced survival and failure to respond to chemo-immunotherapy (MacFarlane *et al.*, 1979). As all the patients with melanoma were in the same stage grouping, increased α subunit levels suggested a more malignant, unresponsive type of melanoma. However, in the premenopausal patients with breast cancer studied here, we are unable to assume that high α levels represent a more malignant tumour; they may merely reflect a greater tumour burden.

The use of α subunit as a tumour marker appears limited. The increases in α concentrations found in the patients with breast cancer were usually moderate and there was considerable overlap in serum α levels between the 3 groups (cancer, benign and controls). Fluctuations of α level during the menstrual cycle reduce the potential of serial measurements of α subunit for detecting early tumour recurrence in premenopausal patients. However, the strong correlation between high α levels and more advanced disease stage in premenopausal patients suggests that α subunit measurement, preferably several samples taken on different days and related to concurrent LH and FSH levels, may be a useful adjunct to more accurate staging. Aids to the staging of breast cancer are needed, as most patients with regional nodes involved at the time of mastectomy already have disseminated disease (Bonnadonna *et al.*, 1976). The correlation of α subunit levels with subsequent response to a hormonal manipulation or chemotherapy may form the basis for further study, in view of the trend towards increased α levels in patients with $RE_C^-$ tumours.

I.A.M. was supported by a grant from the North West Regional Health Authority during the course of this work.

We thank NIAMDD National Pituitary Agency, Baltimore, Maryland for the gift of α TSH and its antiserum. We are grateful to Mrs B. M. Cekalo for typing the manuscript.

### REFERENCES

BARNES, D. M., RIBEIRO, G. G. & SKINNER, L. G. (1977) Two methods for measurement of oestradiol-17β and progesterone receptors in human breast cancer and correlation with response to treatment. *Eur. J. Cancer*, **13**, 1133.

BARNES, D. M., SKINNER, L. G. & RIBEIRO, G. G. (1979) Triple hormone-receptor assay: A more accurate predictive tool for the treatment of advanced breast cancer. *Br. J. Cancer*, **40**, 862.

BONADONNA, G., BRUSAMOLINO, E., VALAGUSSA, P. & 8 others (1976) Combination chemotherapy as an adjuvant treatment in operable breast cancer. *N. Engl. J. Med.*, **294**, 405.

BRAUNSTEIN, G. D., FORSYTHE, A. B., RASOR, J. L., VAN SCOY-MOSHER, M. B., THOMSON, R. W. & WADE, M. E. (1979) Serum glycoprotein hormone alpha subunit levels in patients with cancer. *Cancer*, **44**, 1644.

COVE, D. H., SMITH, S. C. H., WALKER, R. & HOWELL, A. (1979) The synthesis of glycoprotein hormone α subunit by human breast carcinomas. *Eur. J. Cancer*, **15**, 693.

DOSOGNE-GUERIN, M., STALARCZYK, A. & BORKOWSKI, A. (1978) Prospective study of the α and β subunits of human chorionic gonadotrophin in the blood of patients with various benign and malignant conditions. *Eur. J. Cancer*, **14**, 525.

HAGEN, C., GILBY, E. D., MCNEILLY, A. S., OLGAARD, K., BONDY, P. K. & REES, L. H. (1976) Comparison of circulating glycoprotein hormones and their subunits in patients with oat cell carcinoma of the lung and uraemic patients on chronic dialysis. *Acta Endocrinol.*, **83**, 26.

HÄHNEL, R., WOODINGS, T. & VIVIAN, A. B. (1979) Prognostic value of oestrogen receptors in primary breast cancer. *Cancer*, **44**, 671.

HARMER, M. H. (Ed.) (1978) *Classification of malignant tumours, 3rd edition.* Geneva: U.I.C.C.

KAHN, C. R., ROSEN, S. W., WEINTRAUB, B. D., FAJANS, S. S. & GORDON, P. (1977) Ectopic production of chorionic gonadotrophin and its subunits by islet cell tumours. A specific marker for malignancy. *N. Engl. J. Med.*, **297**, 565.

KIANG, D. T., FRENNING, D. H., GOLDMAN, A. I., ASCENSAO, V. F. & KENNEDY, M. D. (1978) Oestrogen receptors and responses to chemotherapy and hormonal therapy in advanced breast cancer. *N. Engl. J. Med.*, **299**, 1330.

KOURIDES, I. A., WEINTRAUB, B. D., RIDGWAY, E. C. & MALOOF, F. (1975) Pituitary secretion of free alpha and beta subunit of human thyrotrophin in patients with thyroid disorders. *J. Clin. Endocrinol. Metabol.*, **40**, 872.

LIPPMAN, M. E., ALLEGRA, J. C., THOMPSON, B. & 7 others (1978) The relation between oestrogen receptors and response rate to cytotoxic chemotherapy in metastatic breast cancer. *N. Engl. J. Med.*, **298**, 1223.

MACFARLANE, I. A., THATCHER, N., SWINDELL, R., BEARDWELL, C. G., HAYWARD, E. & CROWTHER, D. (1979) Serum glycoprotein hormone alpha subunit values and survival in metastatic melanoma patients. *Eur. J. Cancer*, **15**, 1497.

MACFARLANE, I. A., ROBINSON, E. L., BUSH, H. & 4 others (1980*a*) Thyroid function in patients with benign and malignant breast disease. *Br. J. Cancer*, **41**, 478.

MACFARLANE, I. A., BEARDWELL, C. G., SHALET, S. M., DARBYSHIRE, P. J., HAYWARD, E. & SUTTON, M. L. (1980*b*) Glycoprotein hormone α subunit secretion by pituitary adenomas: Influence of external irradiation. *Clin. Endocrinol.*, **13**, 215.

ROSENBERG, E. & BULAT, G. (1979) Immunoreactive α and β subunits of FSH and LH in peripheral blood throughout the menstrual cycle and following stimulation with synthetic gonadotrophin releasing hormone (GnRH). *J. Endocrinol. Invest.*, **2**, 233.

SAY, C. & DONEGAN, W. L. (1974) Invasive carcinoma of the breast: Prognostic significance of tumour size and involved axillary lymph nodes. *Cancer*, **34**, 468.

VAITUKAITIS, J. L., ROSS, G. T., BRAUNSTEIN, G. D. & RAYFORD, P. L. (1976) Gonadotrophins and their subunits: Basic and clinical studies. *Recent Prog. Hormone Res.*, **32**, 289.

WALKER, R. A. (1978) Significance of α subunit HCG demonstrated in breast carcinomas by the immunoperoxidase technique. *J. Clin. Pathol.*, **31**, 245.